# A blended teaching and learning model for family-medicine registrar training at a South African university

**DOI:** 10.4102/phcfm.v16i1.4589

**Published:** 2024-09-26

**Authors:** Ann Z. George, Carien Lion-Cachet, Michele Torlutter, Neetha Erumeda, Deidre Pretorius

**Affiliations:** 1 Centre for Health Science Education, Faculty of Health Sciences, University of the Witwatersrand, Johannesburg, South Africa; 2 North West Province Department of Health, Dr Kenneth Kaunda District Health Services, Klerksdorp, South Africa; 3 Department of Family Medicine, Faculty of Health Sciences, University of the Witwatersrand, Johannesburg, South Africa; 4 Gauteng Department of Health, Johannesburg Metropolitan District Health Services, Johannesburg, South Africa; 5 Gauteng Department of Health, Johannesburg Ekurhuleni Health Services, Johannesburg, South Africa

**Keywords:** family medicine, registrar training, postgraduate training, blended learning, programme development

## Abstract

Effective primary healthcare is essential in developing countries but faces several challenges, including the lack of standardised training across decentralised sites. In response to unsatisfactory registrar examination outcomes in 2013, the Department of Family Medicine at the University of the Witwatersrand in South Africa introduced a blended teaching and learning programme. The aim of the new programme was to level the playing field by providing uniform online resources on a course site on the university’s learning management system. The uniform online resources would be integrated into the teaching programme. A team consisting of the registrar-training-programme coordinator, an educationalist and five family-medicine consultants from different districts began reviewing the curriculum, selecting appropriate resources and developing the course site. The blended programme was developed and implemented using a phased, participatory research action approach, including phases of evaluation and redesign. Since the implementation of the blended-learning programme in 2017, registrar outcomes have improved, but this has not been the only success attributed to the programme. The programme also resulted in an enhanced focus on teaching and learning, especially among those involved in its development. We share the lessons gleaned from our experiences, emphasising the need for adequate training and teamwork if we are to use technology appropriately and effectively to address the difficulties associated with decentralised training in developing countries.

## Introduction

Recent correspondence in the *Lancet* highlighted the importance of primary healthcare in developing countries^1,2^ and the need to ‘address flaws in primary health care, such as difficulty in training and providing professionals, fragile mechanisms for incorporating technologies, and poor infrastructure (including digital infrastructure)’.^1^ Blended learning, which combines face-to-face and online-learning experiences,^3^ is regarded as essential for effective primary healthcare training at decentralised training sites, that is sites distant to tertiary learning institutions.^4^ Blended learning harnesses the flexibility and accessibility of online resources while emphasising their purposeful and meaningful integration with face-to-face training to support learning maximally.^5^

This short report discusses developing and implementing an innovative blended-learning approach to training Family Medicine registrars across the six decentralised training districts affiliated with the University of the Witwatersrand in Johannesburg, South Africa. Four training districts are located in Gauteng Province (Ekurhuleni, Johannesburg Metro, Sedibeng and the West Rand), and two in the Northwest province (Dr Kenneth Kaunda and Dr Ruth Segomotsi Mompati). The idea was to standardise the training, better prepare registrars for the fellowship examinations of the Colleges of Medicine of South Africa, and, ultimately, translate this into better patient care. We highlight the success of the programme and the challenges we encountered.

## The need for a new training programme

Family Medicine was recognised as a specialist qualification in South Africa in 2007.^6^ From 2007 to 2013, the University of the Witwatersrand retained the non-vocational distance-learning programme that had preceded the Health Professions Council of South Africa’s recognition of Family Medicine as a speciality. The registrar-training programme used a theme-based approach. The themes were drawn from the postgraduate family medicine training document developed by the Family Medicine Education Consortium in 2001.^7^ Unsatisfactory registrar outcomes in the 2013 Fellowship examinations, however, urgently necessitated a different training approach. The theme-based programme was replaced in 2014 by the seminar-based triennial cycle. Registrars were assigned topics from the revised postgraduate family medicine training outcomes 2010^7^ and the clinical domains specified by the Fellowship of the College of Family Physicians of South Africa,^8^ thus aiming to cover the clinical and non-clinical topics. The registrars were required to research and present their topic at weekly district-based seminars attended by family physicians from their district. While the seminar-based programme allowed for more structured learning than the previous theme-based approach, its shortcomings meant it would also have to be replaced. The major shortcomings of the triennial cycle were that teaching conducted in the first year would only be taught again in the next cycle, the lack of standardisation of the content taught across districts, and often a failure to cover all the content in the allotted period.

## Developing and implementing the blended-learning programme

**FIGURE 1:**
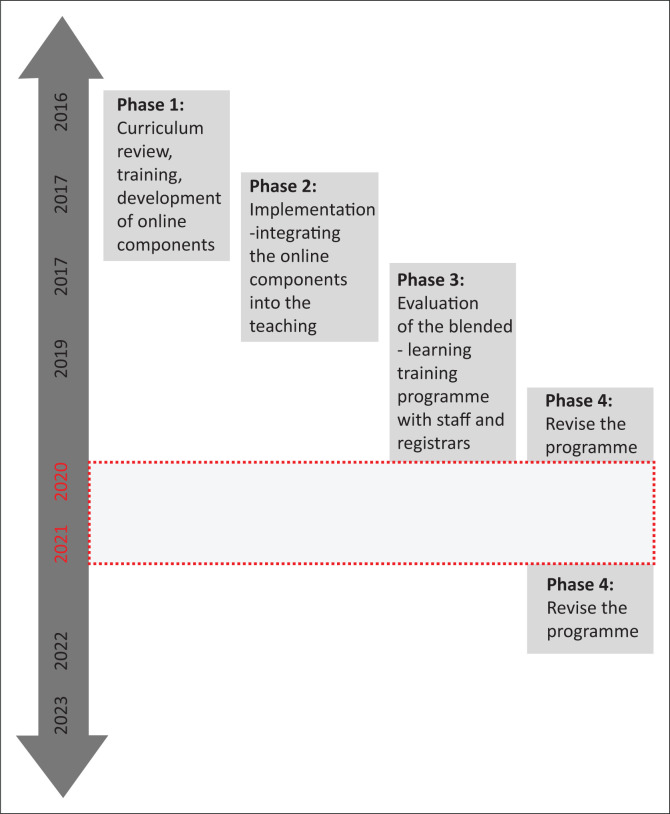
Phases of development of the family medicine, blended-learning, registrar-training programme at the University of the Witwatersrand.

In 2016, a team consisting of the registrar-training programme coordinator, an educationalist, and five family-medicine coordinators from different training districts undertook to replace the triennial cycle with a blended-learning programme. The programme was developed as a participatory-action-research study (HREC (Medical): M170828), using a phased approach (Figure 1). An initial curriculum review was used to identify the core content and supporting resources needed across the vast family-medicine curriculum. The online resources included the most recent clinical guidelines and links to relevant websites. Training the coordinators on using the university’s learning management system (LMS) was prioritised during the first phase. The next step was structuring the course site, uploading the online components, and checking that all documents could be accessed and the links worked.

The implementation phase began with LMS training for supervisors and registrars, which was conducted at the university and in the districts. The focus during this phase was to integrate the online components into teaching through cross-district, integrated sessions during which biopsychosocial and ethical components were linked to clinical domains. After the programme was rolled out, we interviewed supervisors and conducted focus-group discussions with registrars as part of the evaluation phase, which then informed the redesign of the programme. The redesign aligned unit standard or core course content with assignment submissions and sequencing rotations in line with the examination administered 18 months into the programme.

## Challenges and successes of the programme

We faced several challenges when developing the blended-learning programme (Box 1). Phases 1 and 2 were the most challenging, given our time constraints. The new programme had to be rolled out in 2017 to avoid starting a new triennial learning cycle. In addition to contending with the challenges listed in Box 1, including the consultants finding sufficient time to work on the new programme and their different perspectives on what needed to be taught, the development team also had to contend with external challenges. Once the initial hurdle of the lack of training and experience in using the LMS was addressed, a new LMS introduced in 2018 meant that the learning materials had to be transferred to the new system and additional training conducted. In 2020, the coronavirus disease 2019 (COVID-19) halted the evaluation and redesign phases, and the general academic fatigue post-COVID-19 made it difficult to restart the process. Another external challenge related to mandatory courses added by the faculty added to the registrars’ workload post-COVID-19, which required adjustments to the programme.

BOX 1 Some challenges encountered while developing the blended-learning training programme.

**Technical challenges**

- Mastering the University’s learning management system.
- Designing the course layout for easy and logical access.
- Being mindful of copyright considerations when uploading resources.
- Ensuring training for all end-users when not all were equally enthusiastic about the new programme.

**Academic challenges**

- Determining the depth and breadth of the learning material.
- The lack of clinical team members’ experience in programme development.
- Ensuring the clinical guidelines uploaded were current.
- Encouraging more consultants to be involved in the process to divide the workload.
- The impact of the COVID-19 pandemic on the programme in delaying the revision and renewal of the online resources.
- Quality assurance of the programme, especially the content included.

**Teamwork challenges**

- Maintaining momentum despite heavy workloads and service delivery demands.
- Managing interpersonal-relationships and team dynamics.

## Conclusion

Despite the challenges, the new programme has been highly successful. Our pass and throughput rates have improved substantially since introducing the blended programme. Our Fellowship pass rates have ranged between 75% and 100% since 2019, with all students graduating within the allotted time. All four registrars who sat for the Fellowship examinations in 2021 passed on their first attempt,^9^ in stark contrast to none having passed in 2013. The programme’s success is attributed mainly to the unit standards designed to focus the registrars on essential content and direct them to further reading. Scheduled online sessions helped them to translate the content into practical scenarios and encouraged context-specific critical reasoning and systems thinking. Another aspect of the purposeful integration of the online resources across the teaching programme was access to the most relevant clinical guidelines, which reduced the registrars’ uncertainty about which resources to use. The revision of the programme resumed in 2023, with new plans underway to revitalise and expand the blended-learning programme by including, for example, context-specific case scenarios and skills videos. New consultants have been invited to participate, some of whom were taught using the blended-learning programme, thus ensuring the transfer of skills and a potential influx of new ideas.

Developing and implementing a new postgraduate blended-learning programme across decentralised sites is a major undertaking. One of the major strengths of our undertaking was combining different types of expertise, clinical and educational, to maximise our efforts. The primary lesson we share with others planning or embarking on similar programmes is to apportion sufficient time, including time to review your curriculum, so that you have clear and achievable goals with a realistic timeline. In addition, ensure an adequate number of people to distribute the additional workload involved in revising a postgraduate clinical programme, allowing for staff attrition and external influences. Incorporate evaluation and redesign into your planning, including peer and student feedback. Prioritise training for staff and students, especially in the case of blended-learning programmes – technology remains daunting for some.

The authors would like to thank the registrars and district supervisors who participated in the participatory action research study that contributed to the development of the blended learning programme. A special vote of thanks to Dr. Bishop Uwakata for his contributions to the development of the programme and Ms. Silindile Mbatha for her administrative support.

A.G. and D.P. conceptualised the report. A.G. wrote the first draft of the article. D.P. contributed to subsequent drafts. All authors contributed to the development of the article and approved the final version.

Ethical clearance to conduct this study was obtained from the University of the Witwatersrand, Human Research Ethics Committee (HREC) (Medical) (No. M170828).

This work is based on the research supported in part by the National Research Foundation of South Africa (Grant No. 122003).
